# Antioxidant, Pancreatic Lipase Inhibitory, and Tyrosinase Inhibitory Activities of Extracts of the Invasive Plant *Spartina anglica* (Cord-Grass)

**DOI:** 10.3390/antiox10020242

**Published:** 2021-02-04

**Authors:** Geum Jin Kim, Songhee Park, Eonmi Kim, Hyukbean Kwon, Hae-Jin Park, Joo-Won Nam, Seong-Soo Roh, Hyukjae Choi

**Affiliations:** 1 College of Pharmacy, Yeungnam University, Gyeongsan, Gyeongsangbukdo 38541, Korea; kimgeumjin@naver.com (G.J.K.); psh5959@naver.com (S.P.); minnie60@hanmail.net (E.K.); zero9602@gmail.com (H.K.); jwnam@yu.ac.kr (J.-W.N.); 2 Research Institute of Cell Culture, Yeungnam University, Gyeongsan, Gyeongsangbukdo 38541, Korea; 3 Faculty of Herbal Cuisine and Nutrition, Daegu Haany University, Daegu 42158, Korea; hjpark@dhu.ac.kr; 4 Department of Herbology, Daegu Haany University, Daegu 42158, Korea

**Keywords:** *Spartina anglica*, invasive plant, antioxidant activity, tyrosinase inhibition, pancreatic lipase inhibition

## Abstract

Since 2016, the invasive halophyte *Spartina anglica* has been colonizing mudflats along the western coast of South Korea. In order to minimize costs on *S. anglica* expansion management and waste-treatment of collected biomass, the potential application of the collected biomass of *S. anglica* was investigated. Ethanolic extracts and subfractions thereof (hexanes, methylene chloride, ethyl acetate, 1-butanol, and water-soluble) of the aerial and belowground parts of *S. anglica* showed free radical-scavenging [2,2-diphenyl-1-picrylhydrazyl (DPPH), and 2,2’-azino-bis(3-ethylbenzothiazoline-6-sulfonic acid) (ABTS)], tyrosinase inhibitory, and pancreatic lipase inhibitory activities. An ethyl acetate fraction derived from aerial parts (EA-a) showed the most potent radical-scavenging and pancreatic lipase inhibitory activities, whereas tyrosinase inhibition was mainly observed in the methylene chloride soluble fractions (MC-bg) and other lipophilic fractions (ethyl acetate and hexanes layers) obtained from belowground parts. The major EA-a compound isolated and identified was 1,3-di-*O*-*trans*-feruloyl quinic acid (**1**) based on spectroscopic analysis, whereas the two major MC-bg compounds were identified as *p*-hydroxybenzaldehyde (**2**) and *N*-*trans*-feruloyltyramine (**3**). Compounds **1** and **3** scavenged both DPPH and ABTS radicals, whereas **1** and **2** inhibited pancreatic lipase activity. These results indicate that extracts and fractions of *S. anglica* have antioxidant, anti-obesity, and whitening properties with potential pharmaceutical, cosmeceutical, and functional food applications.

## 1. Introduction

*Spartina anglica* C. E. Hubb (Poaceae) is a herbaceous perennial plant, commonly referred to as “cord-grass”, that inhabits coastal areas of southern England and western France [1]. Given its dense roots that can tightly bind coastal mud, *S. anglica* was initially planted with a view toward protecting coastal areas against erosion and has subsequently been used extensively in the reclamation of tidal flats [2]. However, with the emerging awareness of the ecological importance of mudflats, the detrimental effects of *S. anglica* on mudflat ecosystems have become increasingly apparent. In areas colonized by *S. anglica*, the native vegetation is gradually displaced, thereby transforming mudflat habitats into a comparatively ecologically poor monoculture [3]. In addition, the invasion of *S. anglica* disrupts the habitats of mud-inhabiting invertebrates, migratory birds, and breeding fish, while contributing to biochemical modifications, such as hazardous heavy metal pollution [4,5,6]. *Spartina anglica* is accordingly currently classified as an invasive plant with the potential to devastate coastal mudflats [7]. Since the occurrence of *S. anglica* was first reported in the southern parts of Ganghwa Island, South Korea, in 2012, the area colonized by *S. anglica* has rapidly expanded, and the plant has been designated as an “unintroduced species” by the Korean government [8]. Although management to prevent the invasive expansion of *S. anglica* in coastal areas of Korea is considered a high priority, it is labor- and time-intensive. Furthermore, the collected biomass of *S. anglica* is a biological waste that incurs additional waste-treatment costs. To minimize these costs, we attempted to discover a potential use for the collected biomass of *S. anglica* as a source of bioactive compounds for the development of biomedicines and cosmetics.

Reactive oxygen species (ROS) are a byproduct of cell metabolism and are involved in biological processes such as apoptosis and protection of cells against pathogens [9]. However, owing to their high oxidizing property, ROS can damage tissues and organs and are associated with the aging process as well as diverse human diseases such as cancers, atherosclerosis, and diabetes [9]. Natural antioxidants are scavengers of ROS and protect tissues and organs from ROS-derived oxidative stress. Therefore, several industrial markets exist for natural antioxidants as ingredients of functional foods and cosmetics [10]. Tyrosinase is an enzyme related to the hyperpigmentation of human skin, and its inhibitor can be used as a bioactive ingredient in skin-whitening cosmetics [11,12]. Pancreatic lipase hydrolyzes dietary fat into free fatty acids and monoglycerides, and its inhibitor (orlistat) is widely used as an anti-obesity drug in clinical settings [11].

Previous chemical investigations of *S. anglica* have revealed the presence of dimethylsulfoniumpropanoic acid, proline, glycine betaine, and polysaccharides [13,14,15,16]. In addition, algicidal flavonols, such as isorhamnetin-3-*O*-sophoroside-7-*O*-rhamnoside and syringetin-3-*O*-galactoside, and antioxidant flavonols, such as 5,4′-dihydroxy-3′-methoxy-flavonol-3-*O*-glucosyl-(1→6)-glucosyl-7-*O*-rhamnoside, 5-hydroxy-3′-methoxy-flavonol-3-*O*-glucosyl-(1→6)-glucosyl-7-*O*-rhamnoside, and 5-hydroxy-3′,4′-dimethoxy-flavonol-3-*O*-glucosyl-(1→6)-glucosyl-7-*O*-rhamnoside, have been reported [17,18]. Apart from identifying these constituents, the biological activities of extracts of *S. anglica*, particularly the antioxidant activities of compounds derived from different plant parts, are yet to be investigated. In this study, we obtained extracts and fractions from the aerial and belowground parts of *S. anglica* and evaluated these for bioactivities with potential industrial applications, namely, free radical (2,2-diphenyl-1-picrylhydrazyl (DPPH) and 2,2′-azino-bis(3-ethylbenzothiazoline-6-sulphonic acid) (ABTS)) scavenging activities and the inhibition of pancreatic lipase and tyrosinase.

## 2. Materials and Methods

### 2.1. Chemicals and Reagents

All organic solvents used for extraction, liquid–liquid extraction, and normal phase (NP) vacuum liquid chromatography (VLC) (ethanol, hexanes, methylene chloride, ethyl acetate, and 1-butanol) were of extra-pure grade and obtained from Duksan Chemical Co. (Ansan, South Korea). Acetonitrile (high-performance liquid chromatography (HPLC) and liquid chromatography–mass spectrometry grade), methanol (HPLC grade), and formic acid (liquid chromatography–mass spectrometry grade) were obtained from Fisher Chemical (Pittsburgh, PA, USA). Deionized water was collected from a Direct-Q 5 UV water purification system (18 MΩ cm^−1^; Merck KGaA, Darmstadt, Germany). The internal calibrant (methyl 3,5-dinitrobenzoate) for quantitative ^1^H nuclear magnetic resonance, 2,2-diphenyl-1-picrylhydrazyl (DPPH), 2,2′-azino-bis(3-ethylbenzothiazoline-6-sulfonic acid) (ABTS), potassium persulfate, Folin-Ciocalteu’s phenol reagent, gallic acid, tyrosinase, l-3,4-dihydroxyphenylalanine (l-DOPA), kojic acid, porcine pancreatic lipase (Type II), orlistat, 4-morpholinepropanesulfonic acid (MOPS), ethylenediaminetetraacetic acid (EDTA), *p*-nitrophenyl butyrate (*p*-NPB), dimethylformamide (DMF), Tris(hydroxymethyl)aminomethane hydrochloride (Tris-HCl), and absolute ethanol were purchased from Sigma Aldrich Co. (St. Louis, MO, USA). Sodium carbonate was obtained from Daejung Chemicals & Metals Co., Ltd. (Siheung, Korea). Dimethyl sulfoxide (DMSO) was purchased from PanReac Applichem (Darmstadt, Germany). l-Ascorbic acid was purchased from Alfa Aesar (Lancashire, UK).

### 2.2. Plant Materials

In July 2016, specimens of *S. anglica* (30 kg) were collected from a mudflat on Ganghwa Island, Incheon, South Korea (126°27′18″ E, 37°35′36″ N) and were identified by one of the authors (H.C.). A voucher specimen (16K009) has been deposited at the College of Pharmacy, Yeungnam University, Gyeongsan, Korea.

### 2.3. Extraction and Fractionation

The collected plant material was washed and dried at 24 °C under dark conditions for 1 week, and the dried plants were subsequently separated into aerial and belowground parts. Each part (dry wt. 10 kg) was extracted three times with 80% EtOH and dried under reduced pressure, thereby yielding extracts of the aerial (EtOH-a, 3.3 kg) and belowground parts (EtOH-bg, 554 g) parts. These crude extracts were further fractionated using liquid–liquid extraction. A small portion of the EtOH-a extract (1.8 g) was re-suspended in 250 mL of deionized water and extracted three times with 250 mL of hexanes. The combined hexanes-soluble layers of aerial part extracts were evaporated to give a hexanes-soluble fraction (Hex-a, 102 mg). The remaining extract of the aerial parts was extracted three times with methylene chloride and the combined organic layers were dried to give a methylene chloride-soluble fraction of the aerial parts (MC-a, 92 mg). The residual aqueous layer was extracted three times with ethyl acetate and the resulting aqueous layer was further extracted three times with water-saturated 1-butanol. The ethyl acetate, 1-butanol, and aqueous layers were independently lyophilized to give the fractions EA-a (111 mg), BuOH-a (531 mg), and H_2_O-a (1015 mg), respectively. Using the same procedures, a small portion (11.0 g) of the EtOH-bg extract was fractionated to yield the following five sub-fractions: Hex-bg (234 mg), MC-bg (271 mg), EA-bg (196 mg), BuOH-bg (241 mg), and H_2_O-bg (9400 mg).

### 2.4. DPPH Free Radical-Scavenging Activity

DPPH free radical-scavenging properties were evaluated using a previously reported method [19]. Test sample (an extract, a fraction or a compound) in 100 μL of dimethyl sulfoxide (DMSO) (blank: 100 μL of DMSO solution) was added to 100 μL of an ethanolic solution of DPPH (60 μM) in the wells of a 96-well microplate. Ascorbic acid (standard sample) and each sample were prepared at 100 μg/mL. After the sample and the reagents had been mixed gently and incubated for 30 min at room temperature, the absorbance (*A*) at 540 nm was evaluated using a microplate reader (Model Infinite M200 PRO; Tecan, Austria). The free radical-scavenging activity was calculated as a percentage using the equation

DPPH free radical-scavenging activity (%) = [1 − (*A*_sample_/*A*_blank_)] × 100. (1)

### 2.5. ABTS Assay

ABTS radical-scavenging activity was measured using a modified method of Re et al. [20]. The ABTS stock solution was diluted in water to a concentration of 7.4 mM. The ABTS radical was produced by mixing ABTS stock solution with 2.45 mM potassium persulfate and incubating in dark for 12 h at room temperature. The ABTS solution was diluted with ethanol to obtain an absorbance (*A_blank_*) of 0.70 ± 0.02 at 415 nm. After adding 95 μL of diluted ABTS solution to 5 μL of the sample, the mixture was incubated at room temperature for 15 min in dark. The absorbance (*A_sample_*) at 415 nm was measured using a microplate reader (Model Infinite M200 PRO; Tecan, Austria). The blank was prepared using the same procedure with deionized water instead of the sample. The ABTS radical scavenging activity was calculated as a percentage using the Equation (2)

ABTS radical scavenging activity (%) = [1 − (*A*_sample_/*A*_blank_)] × 100 (2)

### 2.6. Tyrosinase Inhibition Assay

The tyrosinase inhibition assay was performed using a modification of the method reported by Nerya et al. [21]. A portion of a sample (20 μL) in DMSO (100 μg/mL) was mixed with 50 μL of 0.1 M potassium phosphate buffer (pH 6.8), to which was added 30 μL of 33 unit/mL tyrosinase and 100 μL of 12 mM l-3,4-dihydroxyphenylalanine (l-DOPA). The solution was mixed gently and the initial absorbance (*A*_initial_) was recorded at 492 nm using a microplate reader. The reaction mixture was then incubated at 37 °C for 4 min and thereafter the final absorbance (*A*_final_) was measured at 492 nm. Kojic acid was used as the reference drug. The tyrosinase inhibition activity was calculated as a percentage using the Equation (3)

Tyrosinase inhibition activity (%) = [1 − (*A*_final_/*A*_initial_)] × 100 (3)

### 2.7. Pancreatic Lipase Inhibition Assay

Pancreatic lipase activity was measured using the method previously reported by Kim et al. [22]. An enzyme buffer was prepared by the addition of 6 μL of porcine pancreatic lipase solution (Sigma-Aldrich, St. Louis, MO, USA) in a buffer (10 mM morpholinepropanesulfonic acid and 1 mM EDTA, pH 6.8) to Tris buffer (169 μL, 100 mM Tris-HCl, and 5 mM CaCl_2_, pH 7.0). Aliquots (20 μL) of each sample and orlistat at 100 μg/mL were then mixed with 175 μL enzyme buffer and incubated for 15 min at 37 °C with 5 μL of the substrate solution (10 mM *p*-NPB (*p*-nitrophenyl butyrate) in dimethylformamide). The enzymatic reaction mixtures were incubated at 37 °C for 35 min. Pancreatic lipase activity was determined by measuring the hydrolysis of *p*-NPB to *p*-nitrophenol, with absorption being recorded at 405 nm using a microplate reader (Model Infinite M200 PRO; Tecan, Austria). Inhibition of lipase activity was expressed in terms of the percentage reduction in absorbance when porcine pancreatic lipase was incubated with the test compounds, and calculated using the following Equation (4)

Inhibition (%) = 100 − [(*B* − *b*)/(*A* − *a*) × 100] (4)

where *A* is the lipase activity without inhibitor, *a* is the negative control without inhibitor, *B* is the lipase activity with inhibitor, and *b* is the negative control with inhibitor. The results are expressed as an average (n = 4).

### 2.8. Measurement of Total Phenol Contents

Total phenol contents were measured using the Folin–Denis method [23]. Samples (20 μL) were mixed with DMSO (1.58 mL) and Folin–Ciocalteu’s phenol reagent (100 μL), and incubated at room temperature for 1 min, followed by the addition of sodium carbonate solution (20% v/v, 300 μL). After incubating the mixture at 20 °C for 2 h, the absorbance was measured at 765 nm using a microplate reader (Model Infinite M200 PRO; Tecan, Austria). Gallic acid was used as the reference material.

### 2.9. Chemical Profiles of the Biologically Active Fractions (EA-a and MC-bg)

Chromatographic profiling of the most active fractions, EA-a and MC-bg, was performed using an Agilent 1260 Infinity high-performance liquid chromatograph equipped with a reversed-phase C18 column (Phenomenex Luna 3μ C18(2) 100 Å, 4.6 × 150 mm). The solvent system used was as follows: solvent A (95% deionized water, 5% acetonitrile with 0.05% formic acid), solvent B (100% acetonitrile with 0.05% formic acid); A:B = 95:5 → 95:5 (2 min) → 0:100 (12.5 min) → 0:100 (15 min) → 95:5 (16 min) → 95:5 (19 min) at a flow rate of 0.7 mL/min. Compounds were detected using diode array detector (DAD: Agilent 1260 Infinity DAD; Agilent Technologies, Santa Clara, CA, USA) and electrospray ionization-mass spectrometry detector (ESI MS: Agilent 6120 single-quadrupole mass spectrometer; Agilent Technologies, Santa Clara, CA, USA).

### 2.10. Isolation of Major Compounds from the Bioactive Fractions (EA-a and MC-bg)

EA-a (6.6 g) was subjected to stepped-gradient NP VLC to yield 14 fractions (EA-a-A–N). Fractions K–M, eluted with 60–30% ethyl acetate in methanol, were found to include compound **1**. After combining the fractions K–M, EA-a-KLM (935 mg) was subjected to normal phase medium-pressure liquid chromatography for the preparation of nine subfractions (EA-a-KLM-1–9). Among these subfractions, EA-a-KLM-6 (476.7 mg), eluted with 70% methylene chloride in 30% methanol, was injected into a preparative high-performance liquid chromatograph equipped with a reversed-phase C18 column (Phenomenex Luna C18; 100 Å, 22.5 × 250 mm). The system used comprised solvent A (95% deionized water, 5% acetonitrile with 0.05% formic acid) and solvent B (100% acetonitrile) (A:B = 82:18) at a flow rate of 6.0 mL/min, with detection at 210 and 254 nm. Compound **1** (55.2 mg, retention time 38.849 min) was accordingly obtained, the structure of which was determined by spectroscopic analysis.

MC-bg (271.1 mg) was subjected to NP VLC, which yielded seven fractions (MC-bg-A–G). A fraction (MC-bg-D, 56.9 mg) containing compounds **2** and **3** was subjected to preparative HPLC using a reversed-phase C18 column (Phenomenex Luna C18, 100 Å, 22.5 × 250 mm). The solvent system used comprised solvent A (100% deionized water) and solvent B (100% acetonitrile) (A:B = 74:26) at a flow rate of 6.0 mL/min, with detection at 210 and 254 nm. The structures of compound **2** (0.6 mg, retention time 13.759 min) and **3** (3.2 mg, retention time 35.612 min) thus obtained were confirmed by spectroscopic analyses.

### 2.11. Quantitative Analysis of the Major Compounds in EA-a and MC-bg

The weight percentage of compound **1** in EA-a and that of compounds **2** and **3** in MC-bg were calculated using quantitative ^1^H nuclear magnetic resonance (qHNMR) with an internal calibrant. EA-a and MC-bg were thoroughly dried in a vacuum chamber and three samples of precisely weighed (10.0 mg) EA-a and MC-bg were prepared, each of which was dissolved in 600 μL of DMSO-*d*_6_ (99.8% D; Euriso-Top, Saclay, France). Methyl 3,5-dinitrobenzoate (precisely weighed: 3.4 mg), dissolved in DMSO-*d*_6_ to give a 5.7 mg/mL solution, was used as an internal calibrant. Aliquots (20 μL) of the internal calibrant were added to each prepared EA-a and MC-bg sample solution, and 600 μL of each sample was transferred to a 5 mm NMR tube. ^1^H NMR spectra were acquired at 25 °C in a 600-MHz Fourier Transform Nuclear Magnetic Resonance device (VNS600; Agilent Technologies, Santa Clara, CA, USA) at the Core Research Support Center for Natural Products and Medical Materials (CRCNM) in Yeungnam University with the following parameters: a calibrated 90° pulse, 64 scans, and a 60-s relaxation delay (D1). Each ^1^H NMR spectrum was manually processed by phasing, baseline correction with a fifth-order polynomial function, and referencing with the residual solvent signal at 2.51 ppm, using Mnova 9.0 (Mestrelab Research SL, Santiago de Compostela, Spain). Further processing was performed by apodization (LB = −0.3 Hz, GF = 0.05) and zero filling (256 K data points). For the quantitative analysis of compounds **1**–**3**, a doublet of the sp2 proton at 6.42 ppm of **1**, a singlet of an aldehyde proton at 9.78 ppm and a doublet of *ortho*-coupled sp2 protons at 7.75 ppm of **2**, and a triplet of the sp3 proton at 2.64 ppm of **3**, as well as a doublet of *meta*-coupled protons at 8.91 ppm of the internal calibrant were used. The integration value of each peak was obtained after peak deconvolution to give precise measurement without interference of peak overlap.

### 2.12. Statistical Analysis

All data are presented as the mean ± standard deviation (SD) and are representative of at least three independent experiments. Statistical comparisons between groups were made using Sigma plot 9.0 statistical software (Systat Software Inc, San Jose, CA, USA).

## 3. Results

### 3.1. Biological Activities of the Extracts and Fractions of S. anglica

The antioxidant potentials of *S. anglica* extracts were evaluated based on the scavenging of DPPH free radicals and ABTS radicals. Both the EtOH-a and EtOH-bg extracts (100 μg/mL) of *S. anglica* were found to possess radical-scavenging activities, as shown in Table 1. Compared with EtOH-bg, the EtOH-a extract showed slightly more potent radical-scavenging activities for both DPPH (18.5 % inhibition) and ABTS (39.4% inhibition) radicals. We found that the fractions EA-a, BuOH-a, and MC-a (100 μg/mL each) also scavenged DPPH (55.9%, 41.7%, and 38.4%, respectively) and ABTS (68.8%, 47.3%, and 58.8%, respectively) radicals. Among the fractions derived from an extract of the belowground parts of *S. anglica*, MC-bg and EA-bg also showed antioxidant activities against DPPH (38.1% and 28.6% inhibition, respectively) and ABTS (50.1% and 44.2% inhibition, respectively) radicals.

Extracts and fractions derived from the aerial and belowground parts of *S. anglica* were also evaluated for the inhibition of tyrosinase, which is a key enzyme in melanin biosynthesis via the oxidation of l-DOPA. We found that the fractions EtOH-a and EtOH-bg (100 μg/mL) inhibited the tyrosinase-induced oxidation of l-DOPA by 30.4% and 29.5%, respectively. Furthermore, whereas EA-a showed slightly more potent activity in the radical scavenging assay, MC-bg, EA-bg, and Hex-bg showed more potent tyrosinase inhibition activities (85.0%, 69.4%, and 67.9% inhibition, respectively) than those did fractions derived from of aerial parts, as shown in Table 1.

With respect to determining anti-obesity potentials, we also examined the inhibitory effects of extracts and fractions on pancreatic lipase activity (Table 1). Both EtOH-a and EtOH-bg (100 μg/mL) were found to inhibit pancreatic lipase activities (47.7% and 49.5%, respectively), whereas among the different fractions, EA-a showed the most potent inhibitory activity (51.2% inhibition at 100 μg/mL).

The activities of phytochemical antioxidants tend to be attributed primarily to phenols, and thus we also determined the total phenol contents of the examined extracts and fractions to assess the contribution of phenolic compounds to the antioxidant activities of the samples as shown in Table 2. Among the different fractions, EA-a was found to have the highest phenolic content (155.28 mg/g), with fractions EA-bg, MC-bg and MC-a also containing notable levels of phenolic compounds at concentrations of 105.62, 68.26, and 55.18 mg/g, respectively.

Two bioactive fractions EA-a and MC-bg were selectively analyzed by LC-ESI-MS and compared with an in-house UV spectra library (Figures S1 and S2). Compound **1** was observed as a major component in the HPLC profile (210 and 254 nm) of EA-a, whereas compounds **2** and **3** were found to be the two major components of MC-bg (210 and 254 nm).

### 3.2. Structural Determination of the Major Compounds of Bioactive Fractions

**Figure 1:**
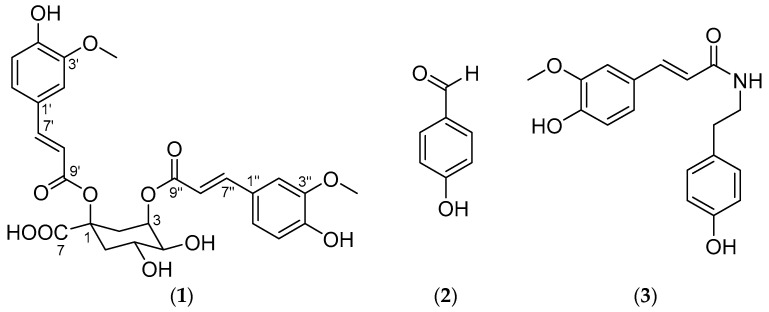
Chemical structures of compounds **1** to **3**; (**1**) 1,3-di-*O*-*trans*-feruloyl-(−)-quinic acid, (**2**) *p*-hydroxybenzaldehyde, and (**3**) *N*-*trans*-feruloyltyramine.

Compound **1** was isolated based on preparative reverse-phase HPLC, and its chemical structure was identified from spectroscopic analyses of 1D and 2D NMR data (Table 3 and Figure S3) using MS data as shown in Figure 1. The ^1^H NMR spectrum of **1** exhibited four vinyl proton resonances at 7.54 (1H, d, *J* = 15.9, H-7″), 7.53 (1H, d, *J* = 15.9, H-7′), 6.26 (1H, d, *J* = 15.9, H-8′), and 6.16 (1H, d, *J* = 15.9, H-8″). The geometries of two double bonds were determined as *trans* by comparison of the coupling constants. The proton resonances at 6.93 (1H, d, *J* = 1.9, H-2′), 6.88 (1H, dd, *J* = 8.2, 1.9, H-6″), 6.83 (1H, d, *J* = 1.9, H-2″), 6.75 (1H, dd, *J* = 8.2, 1.9, H-6′), 6.67 (1H, d, *J* = 8.2, H-5″), and 6.57 (1H, d, *J* = 8.1, H-5′) indicated two sets of 1,3,4-trisubstituted benzene rings. The characteristic proton signals at 5.36 (1H, dt, *J* = 3.6, 3.2, H-3), 4.28 (1H, ddd, *J* = 11.3, 9.6, 4.5, H-5), 3.64 (1H, dd, *J* = 9.6, 3.6, H-4), 2.97 (1H, dt, *J* = 16.2, 3.2, H-2_eq_), 2.54 (1H, ddd, *J* = 13.6, 4.5, 3.2, H-6_eq_), 2.31 (1H, dd, *J* = 16.2, 3.2, H-2_ax_), and 1.84 (1H, dd, *J* = 13.6, 11.3, H-6_ax_) were assigned as a quinic acid moiety based on their multiplicities and splitting patterns [24]. The small coupling constant between δ_H_ 2.97 (1H, dt, *J* = 16.2, 3.2, H-2_eq_) and 2.54 (1H, ddd, *J* = 13.6, 4.5, 3.2, H-6_eq_) was revealed as long-range coupling through 1,3-diequatorial interaction (W-coupling) and H-2_eq_ and H-6_eq_ were both indicated to be equatorial. This corresponds to the ^1^H-^1^H coupling constants of quinic acid rather than that of *epi*-quinic acid. On the basis of these observations, the structure of **1** was predicted to be a quinic acid derivative attached to two phenyl propanoids.

The additional ^13^C NMR signals for C-1 (δ_C_ 81.12) and C-7 (δ_C_ 174.58) were assigned by heteronuclear multiple bond correlation (HMBC) from H-2, 3, 6 to C-1 and H-2, 6 to C-7, respectively. The HMBC correlations from 3′-*O*-Me to C-3 and from 3′-*O*-Me to C-3′ indicated that the two phenyl propanoids are both ferulate. The connection of one feruloyl unit was determined by HMBC correlation from H-3 to C-9″. The position of the other feruloyl group was confirmed by the characteristic downfield chemical shift at δ_C_ 81.12 (C-1). On the basis of these observations, the planar structure of **1** was confirmed as 1,3-di-*O*-*trans*-feruloyl quinic acid [24]. The specific rotation of **1** was measured as −9.7, which corresponds to the specific rotation of (−)-quinic acid rather than that of (+)-quinic acid. Therefore, we confirmed the identity of compound **1** as 1,3-di-*O*-*trans*-feruloyl-(−)-quinic acid with an absolute configuration of 1*R*, 3*R*, 4*S*, 5*R* (1*α*, 3*α*, 4*α*, 5*β*).

Compound **2** was identified as *p*-hydroxybenzaldehyde based on the characteristic UV spectrum and *m/z* value of 121 [M-H]^−^ in the negative ion detection mode along with NMR data (Supplementary Materials, Figure S4 and Table S1), whereas compound **3** was identified as *N*-*trans*-feruloyltyramine based on an interpretation of spectroscopic data, including NMR and MS spectra (Supplementary Materials, Figures S2 and S5, and Table S2) [25].

### 3.3. Quantitative Analysis of Compounds ***1*** to ***3*** in EA-a and MC-bg

Quantitative analysis of the isolated compounds was accomplished based on absolute qHNMR using an internal calibrant. Recently, qHNMR has been established as a standard scientific method for determination of purity and for quantitative analysis applied to the International Conference on Harmonization (ICH) and the United States Pharmacopeia (USP). LC-ESI-MS data revealed that compound **1** was the major component of EA-a, and the weight percentage of **1** in EA-a was calculated using the qHNMR method (Supplementary Materials, Figure S6 and Table S6). Likewise, we calculated the weight percentages of compounds **2** and **3** in MC-bg (Supplementary Materials, Figure S7 and Table S7). Notably, we detected a slight change in the ^1^H NMR chemical shifts of **1** in the extract, and the assignment of the ^1^H NMR signal of compound **1** in EA-a was confirmed by performing a spiking experiment. The weight percentage of compound **1** in EA-a was calculated to be 4.64 ± 0.26% (CV 0.06), whereas those of compounds **2** and **3** in MC-bg were calculated as 0.87 ± 0.01% (CV 0.01) and 4.61 ± 0.03% (CV 0.01), respectively.

### 3.4. Biological Activities of Compounds ***1*** to ***3***

**Figure 2:**
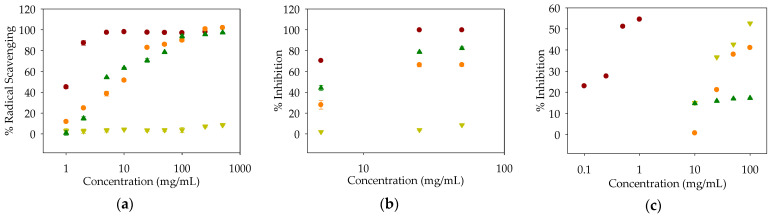
Dose-dependent activities of compounds **1** to **3** [control (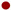); **1** (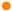); **2** (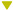); **3** (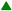)]: (**a**) DPPH radical-scavenging activity (control: l-ascorbic acid); (**b**) ABTS radical-scavenging activity (control: l-ascorbic acid); (**c**) Pancreatic lipase activity (control: orlistat).

Compounds **1**–**3** were isolated as the major bioactive compounds in the fractions obtained and were evaluated in antioxidant assays (Figure 2). While compound **2** showed no antioxidant activity up to 500 μg/mL (4.1 mM), we found that compounds **1** and **3** scavenged both DPPH and ABTS radicals (Table 4). Compound **1**, a major compound of EA-a, was found to scavenge DPPH and ABTS radicals with IC_50_ values of 17.50 and 30.06 μM, respectively. Similarly, compound **3** showed antioxidant activities against DPPH and ABTS with IC_50_ values of 14.90 and 26.14 μM, respectively (Supplementary Materials, Tables S3 and S4).

In addition to the aforementioned antioxidant activities, we observed that compounds **1** and **2** inhibited pancreatic lipase (Table 4), although the inhibitory activity tended to be relatively weak, with IC_50_ value of 131.72 and 86.71 μg/mL (241.91 and 710.02 μM), respectively.

## 4. Discussion

In this study, we found that the extracts and fractions of *S. anglica* exhibited free radical scavenging, pancreatic lipase inhibitory, and tyrosinase inhibitory activities. Fraction EA-a showed the most potent free radical scavenging and pancreatic lipase inhibitory activities, while MC-bg possessed the most potent tyrosinase inhibitory activity.

Chemical investigation of the bioactive fractions led to the isolation and identification of major compounds, 1,3-di-*O*-*trans*-feruloyl-(−)-quinic acid (compound **1**, 4.64%) from EA-a, *p*-hydroxybenzaldehyde (compound **2**, 0.87%) from MC-bg, and *N*-*trans*-feruloyltyramine (compound **3**, 4.61%) from MC-bg. Compound **1** showed free radical (DPPH and ABTS) scavenging activity and moderately inhibited pancreatic lipase activity, compound **2** showed moderate pancreatic lipase inhibitory activity, and compound **3** showed DPPH and ABTS radical scavenging activities.

Although the antioxidant activities of compound **3** were higher than those of compound **1**, we found that EA-a showed more potent antioxidant activity than MC-bg, indicating that unidentified minor components might have additive or synergistic effects on the antioxidant activity of EA-a. Notably, although compound **1** was identified as the major constituent of EA-a, we found that fraction EA-a showed a slightly more potent lipase inhibitory activity than compound **1**, indicating that the lipase inhibitor activity of EA-a is not solely derived from compound **1**.

Likewise, at 100 μg/mL, fraction MC-bg and compound **2** showed pancreatic lipase inhibitory activities of 33.3% and 52.65%, respectively (Supplementary Materials, Table S5). Thus, we speculate that in addition to compound **2**, there are trace components or components contributing to the lipase inhibitory activity of MC-bg.

Compounds **1**–**3** have been shown to have an inhibitory effect on the enzyme tyrosinase. For example, compound **1** isolated from the pollen of *Calendula officinalis* can inhibit the activity of tyrosinase against monophenolase and diphenolase, with IC_50_ values of 44.6 ± 1.5 and 134.4 ± 4.8 μg/mL, respectively [26]. Compound **2** isolated from cumin, a food spice, is a non-competitive tyrosinase inhibitor with an IC_50_ of 820 μM [27,28], whereas compound **3** at 100 μM shows 49 ± 4% inhibition of human melanocyte tyrosinase [29]. Furthermore, compound **3** isolated from a stem extract of *Aurea helianthus* can downregulate microphthalmia-associated transcription factor, tyrosinase, and tyrosinase-related proteins in the inhibition of melanogenesis [30].

The major compounds **1**–**3** derived from bioactive fractions of *S. anglica* in this study are reported herein for the first time from this plant, although they have previously been reported from other plants as tyrosinase inhibitors with skin-whitening potential. The antioxidant activities of sulfur-containing organic acids; amino acids such as proline and glycine betaine, polysaccharides; and algicidal flavonols isolated from extracts of *S. anglica* have also been reported [17,18]. However, to the best of our knowledge, the present study is the first to investigate the radical scavenging, tyrosinase inhibitory, and pancreatic lipase inhibitory activities of sub-fractions of *S. anglica* extracts.

The biological activities and phytochemical composition of plants vary by cultivation region and period, harvesting time, and processing of plant materials [31,32]. Because our results were obtained using the biomass collected in 2016, further studies are needed to investigate the seasonal variation in biological activities and phytochemical composition of *S. anglica*.

Furthermore, there are several reports on the hormetic effects of phytochemicals and herbal medicines [33,34]. Therefore, the hormetic dose–response mechanisms of the extracts and phytochemicals of *S. anglica* and their indirect effects on in vitro/ in vivo bioactivities need to be studied for the industrial development of this plant.

## 5. Conclusions

The biological activities of the extracts and fractions of the invasive plant *S. anglica* were investigated. EtOH-a and EtOH-bg were found to possess radical (DPPH and ABTS) scavenging, tyrosinase inhibitory, and pancreatic lipase inhibitory activities. Among the sub-fractions, the EA-a fraction showed the most potent radical scavenging properties and pancreatic lipase inhibition. The major constituent of EA-a was identified as 1,3-di-*O*-*trans*-feruloyl quinic acid (**1**) with radical scavenging and pancreatic lipase-inhibiting properties. The MC-bg fraction was found to inhibit l-DOPA oxidation. The two major compounds of MC-bg were identified as *p*-hydroxybenzaldehyde (**2**), and *N*-*trans*-feruloyltyramine (**3**), which have been previously reported as tyrosinase inhibitors. Our results indicate that the extracts and fractions of the invasive plant *S. anglica* are potential sources of antioxidants and skin-whitening and anti-obesity agents that could have beneficial applications in the future development of biomedicines, health supplements, and cosmetics.

## Figures and Tables

**Table 1:** Bioactivities of the extracts and fractions of *Spartina anglica* at 100 μg/mL.

Sample	Radical-Scavenging Activity Against DPPH (%)	Radical-Scavenging Activity Against ABTS (%)	Inhibition of l-DOPA Oxidation (%)	Inhibition of Pancreatic Lipase (%)
Control	98.5 ± 0.2 ^1^	99.5 ± 0.3 ^1^	98.6 ± 0.4 ^2^	67.8 ± 0.0 ^3^
EtOH-a	18.5 ± 4.4	39.4 ± 0.4	30.4 ± 1.0	47.7 ± 0.4
Hex-a	2.0 ± 0.6	5.9 ± 0.5	36.8 ± 0.6	36.6 ±0.3
MC-a	38.4 ± 2.7	58.8 ± 0.8	38.2 ± 1.5	38.4 ± 0.5
EA-a	55.9 ± 0.6	68.8 ± 0.3	43.9 ± 0.5	51.2 ± 0.5
BuOH-a	41.7 ± 1.4	47.3 ± 0.3	42.5 ± 1.2	32.7 ± 0.7
H_2_O-a	8.3 ± 1.8	11.8 ± 0.4	44.0 ± 1.6	22.7 ± 0.8
EtOH-bg	11.1 ± 1.4	19.6 ± 0.5	29.5 ± 0.5	49.5 ± 0.5
Hex-bg	6.7 ± 1.2	10.2 ± 0.7	67.9 ± 0.3	46.0 ± 1.2
MC-bg	38.1 ± 1.5	50.1 ± 0.4	85.0 ± 1.8	33.3 ± 0.8
EA-bg	28.6 ± 1.1	44.2 ± 1.2	69.4 ± 0.8	38.6 ± 0.9
BuOH-bg	5.0 ± 6.2	12.2 ± 0.2	43.4 ± 0.6	0.0 ± 0.7
H_2_O-bg	− 0.4 ± 0.4	3.0 ± 0.7	41.6 ± 0.3	26.4 ± 0.6

^1^l-Ascorbic acid. ^2^ Kojic acid. ^3^ Orlistat.

**Table 2:** Measurement of total phenol contents of the extracts and fractions of *Spartina anglica*.

Sample	Total Phenols (mg/g) ^1^
EtOH-a	24.91 ± 0.11
Hex-a	15.60 ± 0.14
MC-a	55.18 ± 0.13
EA-a	155.28 ± 0.30
BuOH-a	33.03 ± 0.13
H_2_O-a	24.94 ± 0.10
EtOH-bg	19.56 ± 0.06
Hex-bg	15.12 ± 0.05
MC-bg	68.26 ± 0.10
EA-bg	105.62 ± 0.36
BuOH-bg	13.87 ± 0.07
H_2_O-bg	11.69 ± 0.08

^1^ Coefficient of variation (CV) value is less than 1.0.

**Table 3:** NMR data of 1,3-di-*O*-*trans*-feruloyl-(−)-quinic acid (**1**) in CD_3_OD.

Unit and Position	δ_C_ (62.5 MHz)	δ_H_ (600 MHz)
(-)-Quinic acid		
1	81.12 ^1^, C	
2	32.74, CH_2_	2.31 (1H, dd, *J* = 16.2, 3.2, H-2_ax_) 2.97 (1H, dt, *J* = 16.2, 3.2, H-2_eq_)
3	73.21, CH	5.36 (1H, dt, *J* = 3.6, 3.2)
4	75.31, CH	3.64 (1H, dd, *J* = 9.6, 3.6)
5	67.80, CH	4.28 (1H, ddd, *J* = 11.3, 9.6, 4.5)
6	41.46, CH_2_	1.84 (1H, dd, *J* = 13.6, 11.3, H-6_ax_) 2.54 (1H, ddd, *J* = 13.6, 4.5, 3.2, H-6_eq_)
7	174.58 ^1^, C	
1-*O*-feruloyl		
1′	127.39, C	
2′	111.51, CH	6.93 (1H, d, *J* = 1.9)
3′	150.63, C	
4′	149.19, C	
5′	116.39, CH	6.57 (1H, d, *J* = 8.2)
6′	123.79, CH	6.75 (1H, dd, *J* = 8.2, 1.9)
7′	147.45, CH	7.53 (1H, d, *J* = 15.9)
8′	115.58, CH	6.26 (1H, d, *J* = 15.9)
9′	168.74, C	
3′-OCH_3_	56.18, CH_3_	3.67 (3H, s)
3-*O*-feruloyl		
1″	127.43, C	
2″	111.57, CH	6.83 (1H, d, *J* = 1.9)
3″	150.35, C	
4″	149.02, C	
5″	116.28, CH	6.67 (1H, d, *J* = 8.2)
6″	124.13, CH	6.88 (1H, dd, *J* = 8.2, 1.9)
7″	146.93, CH	7.54 (1H, d, *J* = 15.9)
8″	115.70, CH	6.16 (1H, d, *J* = 15.9)
9″	167.75, C	
3″-OCH_3_	56.08, CH_3_	3.60 (3H, s)

^1^ Assigned by HMBC data.

**Table 4:** Bioactivities of compounds **1** to **3**.

Sample	Radical-Scavenging Activity Against DPPH IC_50_, μg/mL (μM)	Radical Scavenging Activity Against ABTS IC_50_, μg/mL (μM)	Inhibition of Pancreatic Lipase IC_50_, μg/mL (μM)
Control	1.17 (6.64) ^1^	3.50 (19.87) ^1^	0.49 (0.98) ^3^
**1**	9.53 (17.50)	16.37 (30.06)	131.72 (241.91)
**2**	- ^2^	- ^2^	86.71 (710.02)
**3**	4.67 (14.90)	8.19 (26.14)	- ^2^

^1^l-Ascorbic acid. ^2^ No IC_50_ value calculated.^3^ Orlistat.

## Data Availability

Data is included within the article and the supplementary materials.

## Supplementary Materials

The following data are available online at https://www.mdpi.com/2076-3921/10/2/242/s1, Figure S1: LC-ESI-MS data of EA-a; Figure S2: LC-ESI-MS data of MC-bg; Figure S3: 1D NMR spectra of compound **1** in CD_3_OD; Figure S4: 1D NMR spectra of compound **2** in CD_3_OD; Figure S5: 1D NMR spectra of compound **3** in CD_3_OD; Figure S6: qHNMR spectra of compounds **1** in EA-a with an internal standard; Figure S7: qHNMR spectrum of compounds **2** and **3** in MC-bg with an internal standard; Table S1: NMR data of *p*-hydroxybenzaldehyde (**2**) in CD_3_OD; Table S2: NMR data of *N*-*trans*-feruloyltyramine (**3**) in CD_3_OD; Table S3: DPPH radical-scavenging activity of **1**–**3**; Table S4: ABTS radical-scavenging activity of **1**–**3**; Table S5: Pancreatic lipase inhibition by **1**–**3**; Table S6: Spiked qHNMR data of compound **1** in EA-a; Table S7: qHNMR data of compounds **2** and **3** in MC-bg.
