# Supplementary material for: Antioxidant, Pancreatic Lipase Inhibitory, and Tyrosinase Inhibitory Activities of Extracts of the Invasive Plant Spartina anglica (Cord-Grass)
Source: Antioxidants (Basel). 2021 Feb 4;10(2):242. doi: 10.3390/antiox10020242 (PMC7914639; doi:10.3390/antiox10020242)
Supplement: Supplementary file 1 [file antioxidants-10-00242-s001.pdf]

antioxidants

Supplementary Material

Antioxidant, Pancreatic Lipase Inhibitory, and Tyrosinase Inhibitory Activities of Extracts of the Invasive Plant *Spartina anglica* (Cord-grass)

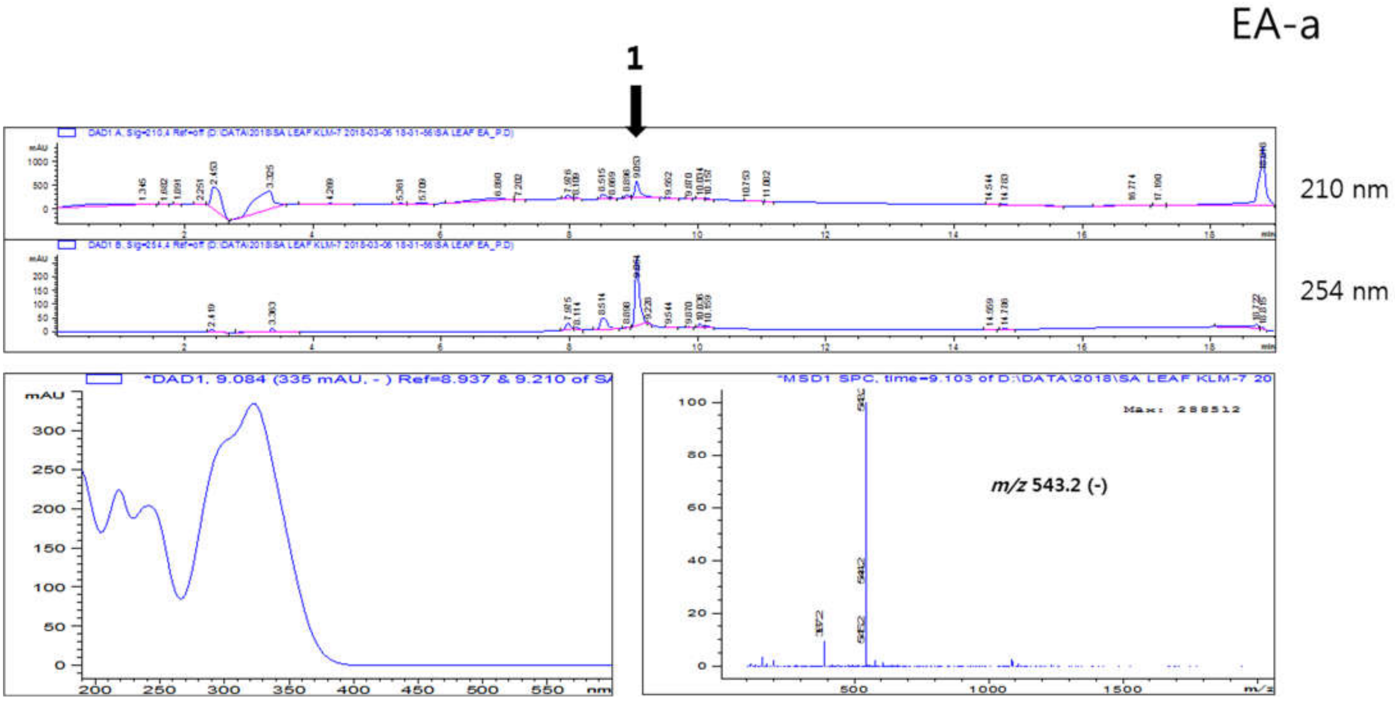

Figure S1. LC-ESI-MS data of EA-a.

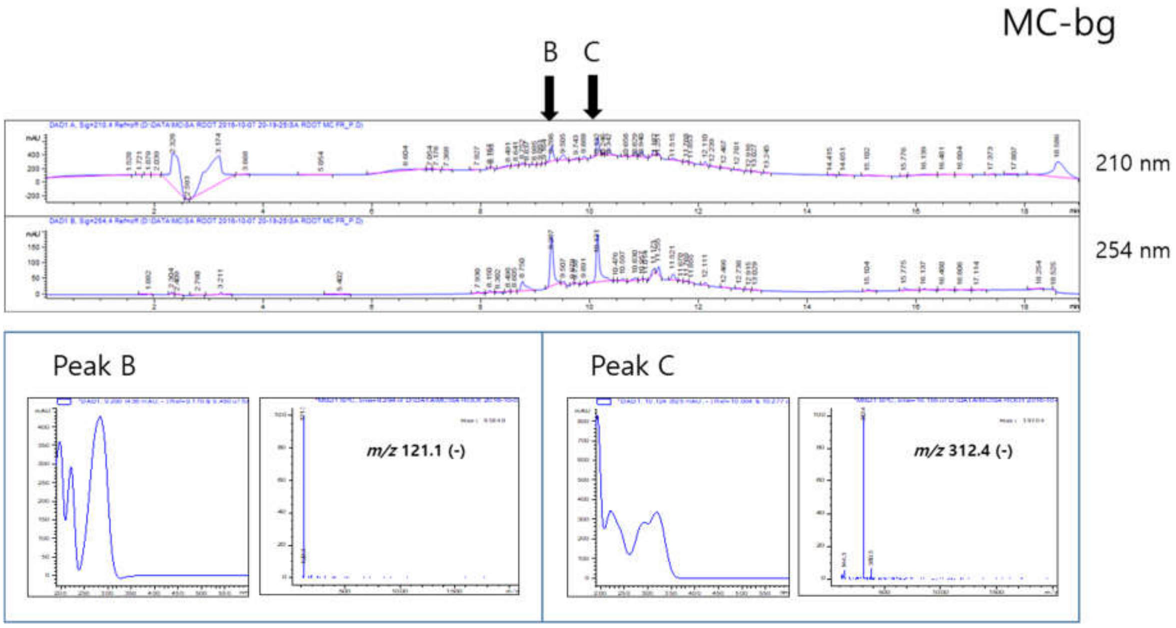

Figure S2. LC-ESI-MS data of MC-bg.

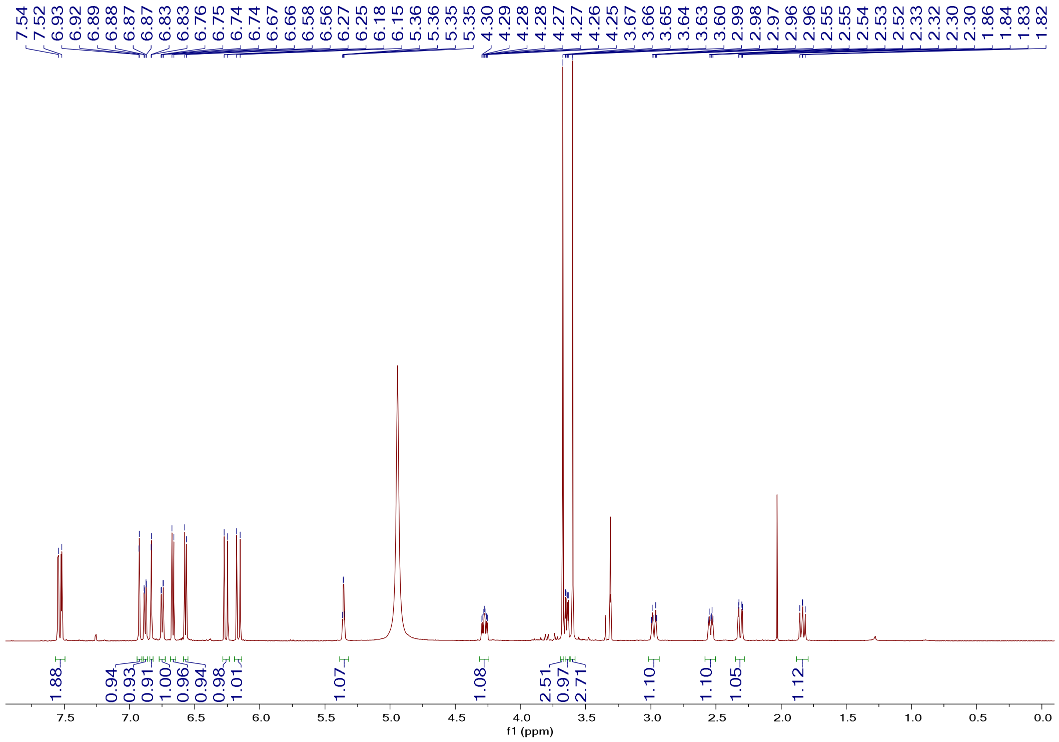

The ^{1}H NMR spectrum of **1** (600 MHz)

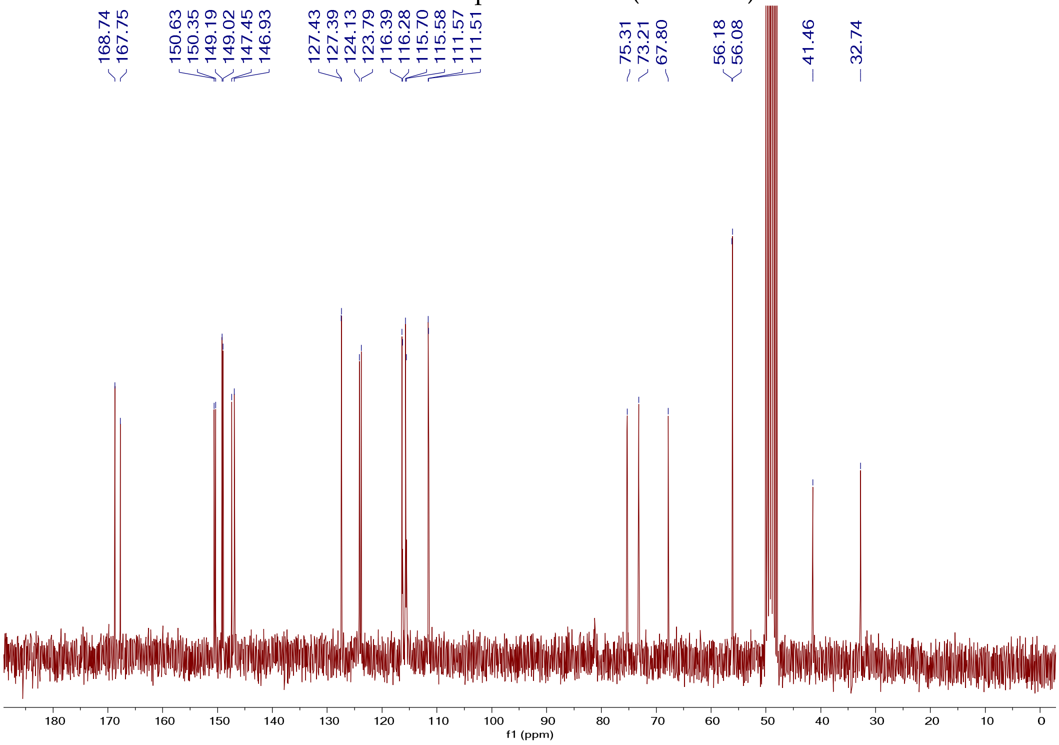

The ^{13}C NMR spectrum of **1** (62.5 MHz)

Figure S3. 1D NMR spectra of compound **1** in CD_3OD.

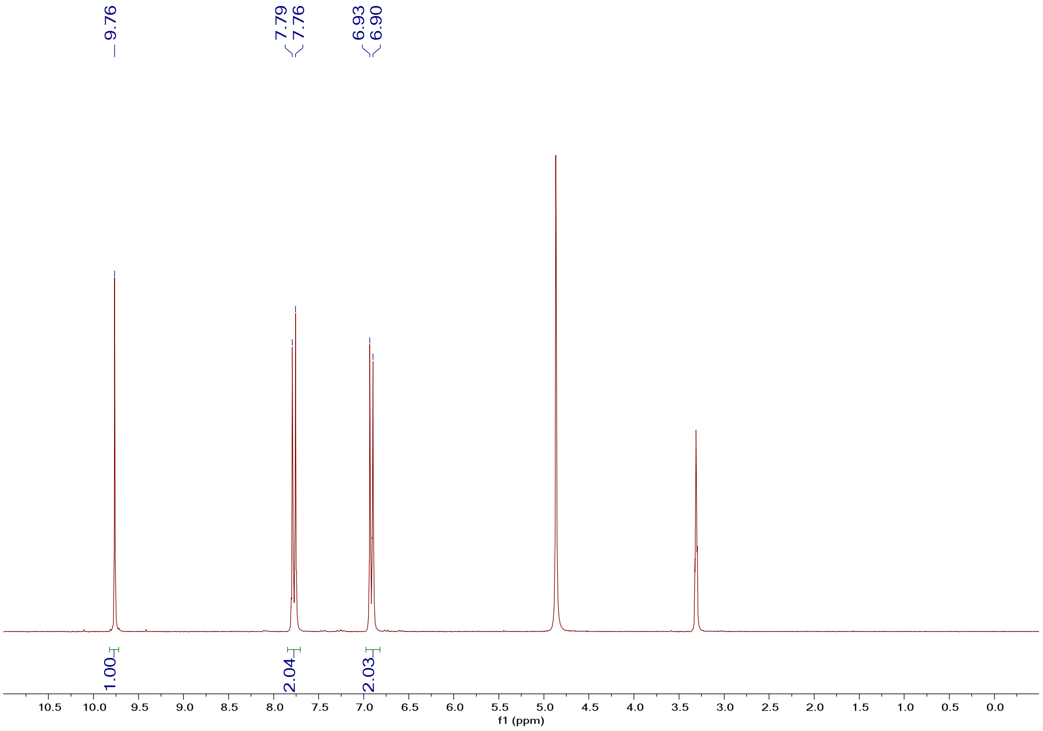

The ^{1}H NMR spectrum of **2** (250 MHz)

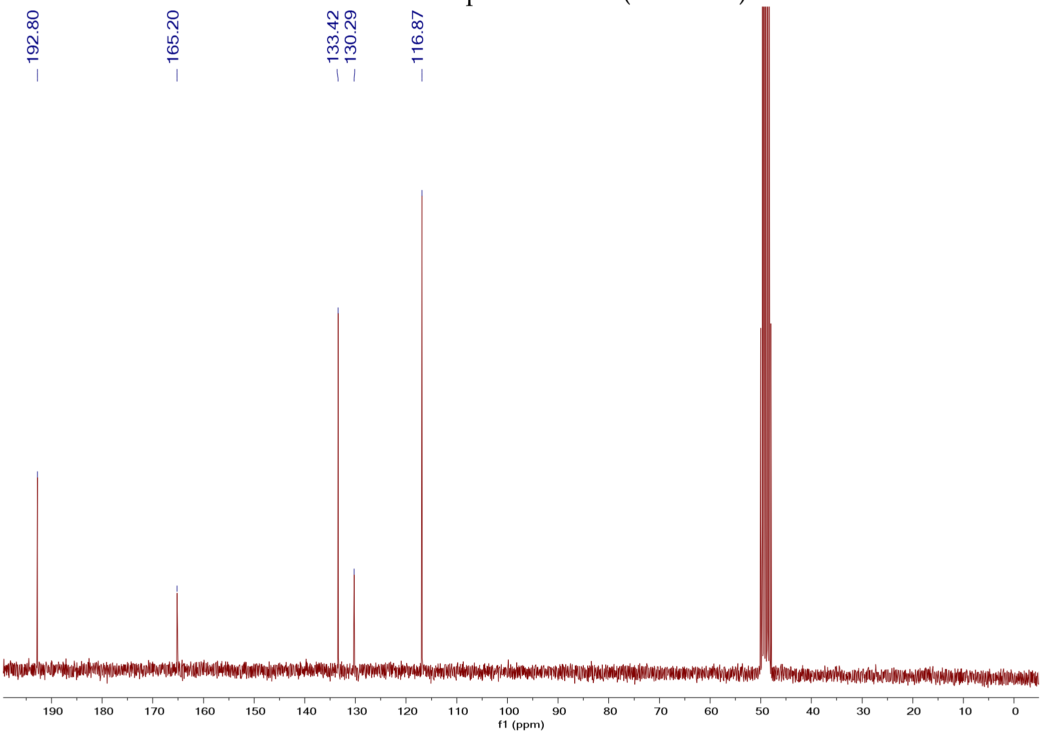

The ^{13}C NMR spectrum of **2** (62.5 MHz)

Figure S4. 1D NMR spectra of compound **2** in CD_3OD.

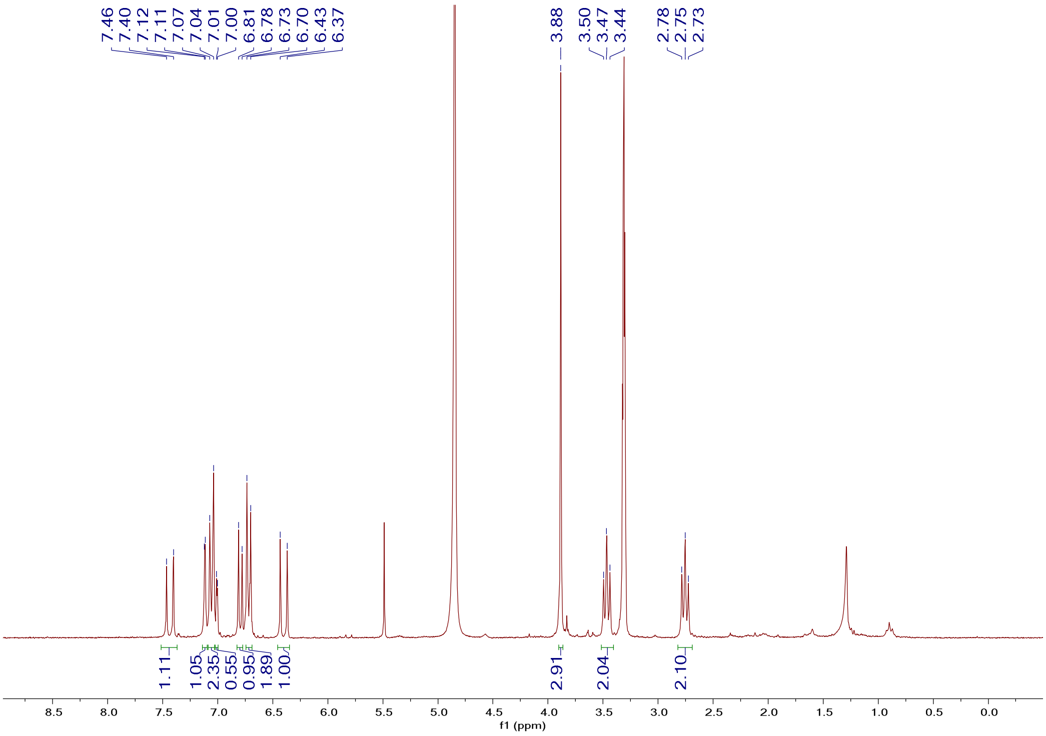

The ^{1}H NMR spectrum of **3** (250 MHz)

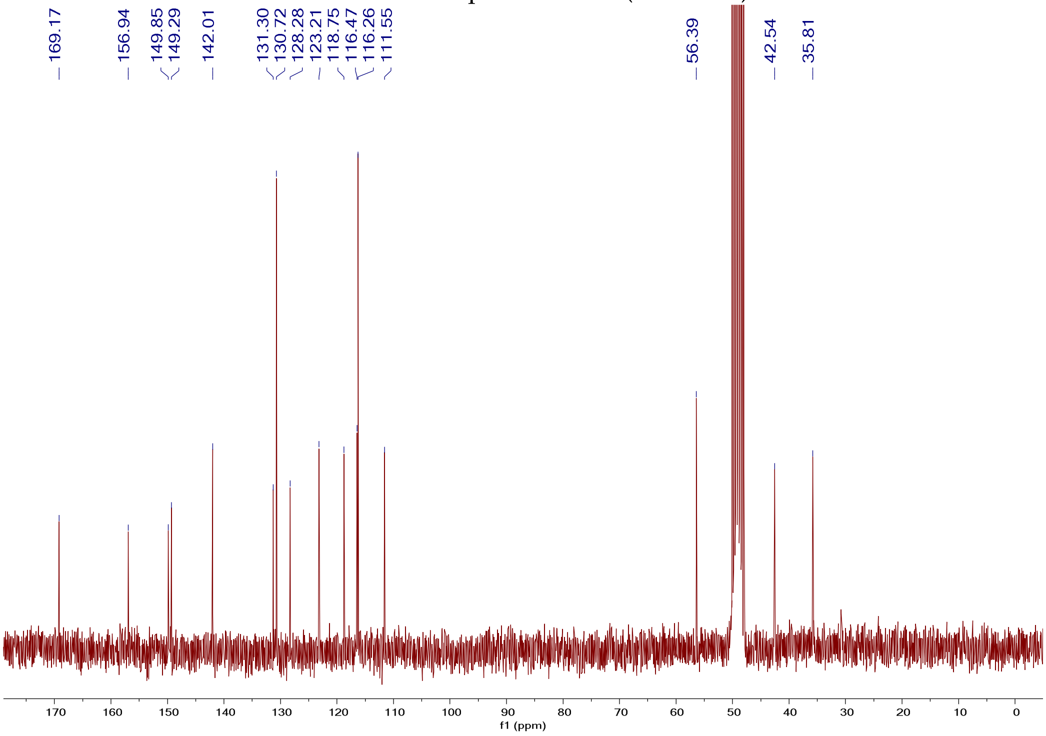

The ^{13}C NMR spectrum of **3** (62.5 MHz)

Figure S5. 1D NMR spectra of compound **3** in CD_3OD.

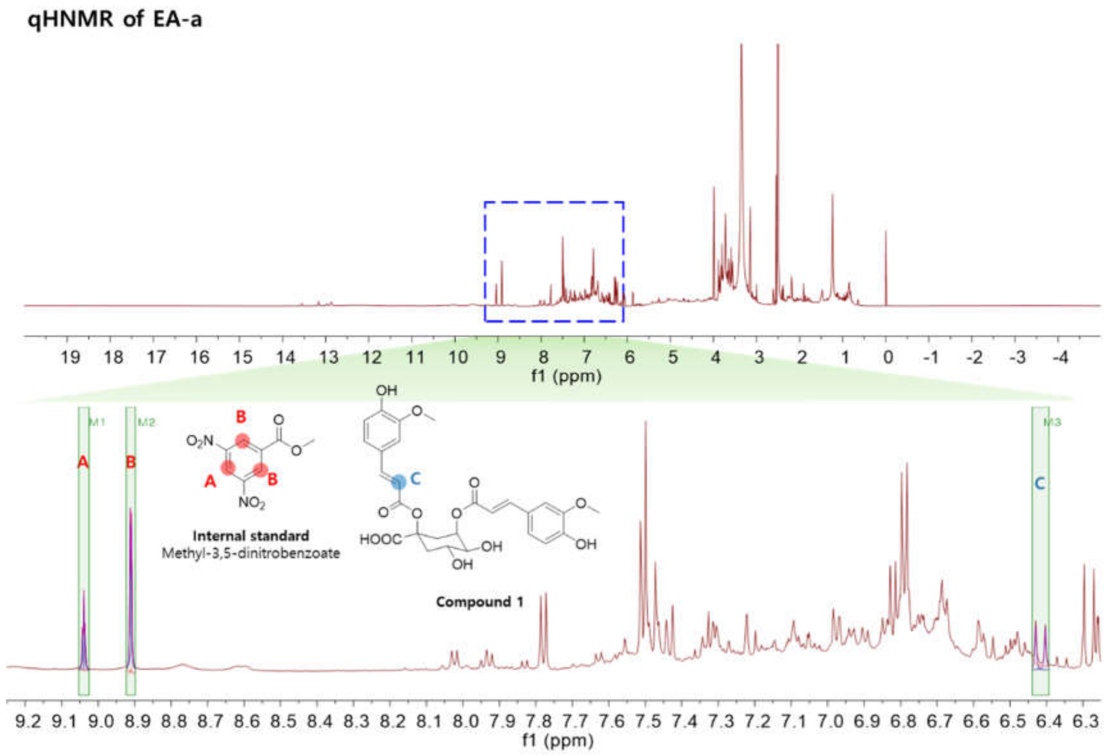

Figure S6. qHNMR spectrum of compound **1** in EA-a with an internal standard.

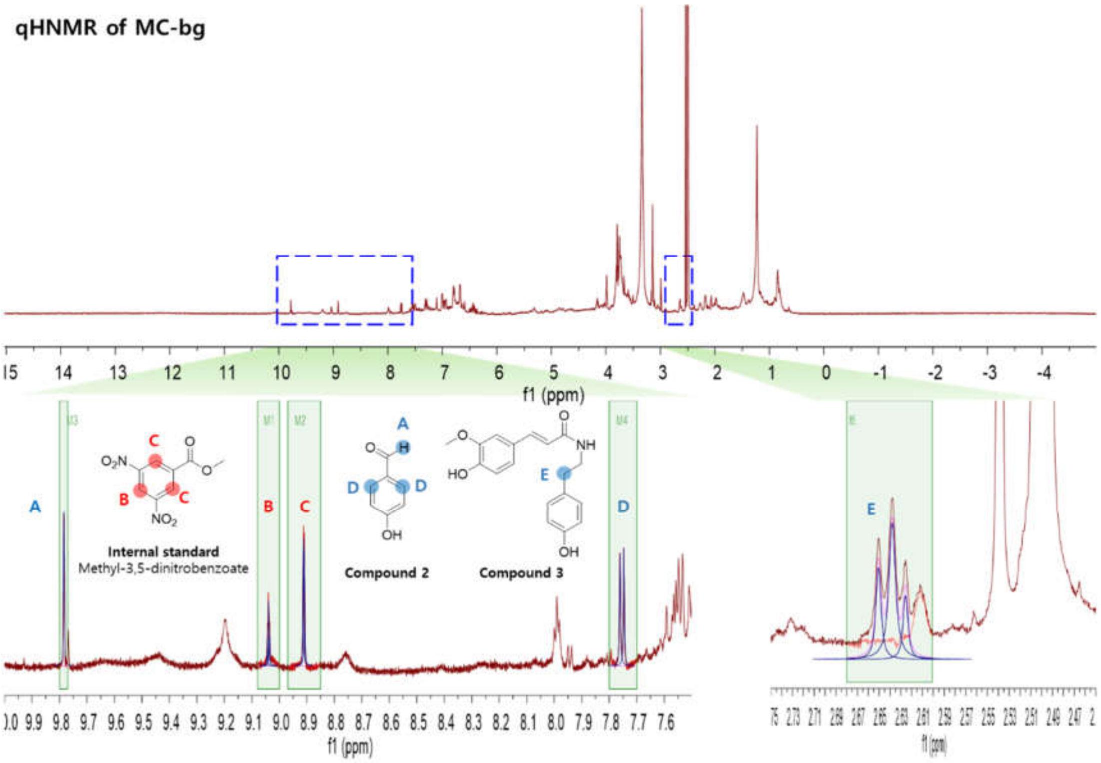

Figure S7. qHNMR spectrum of compounds **2** and **3** in MC-bg with an internal standard

Table S1. NMR data of *p*-hydroxybenzaldehyde (**2**) in CD_3OD.

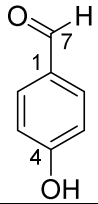

	δC [1]	δH [2]
1	130.29, C	
2	133.42, CH	7.77 (1H, d, *J* = 8.7)
3	116.87, CH	6.91 (1H, d, *J* = 8.7)
4	165.20, C	
5	116.87, CH	6.91 (1H, d, *J* = 8.7)
6	133.42, CH	7.77 (1H, d, *J* = 8.7)
7	192.80, CH	9.76 (1H, s)

[1] Measured in CD_3OD with 62.5 MHz. [2] Measured in CD_3OD with 250 MHz.

Table S2. NMR data of *N-trans*-feruloyltyramine (**3**) in CD_3OD.

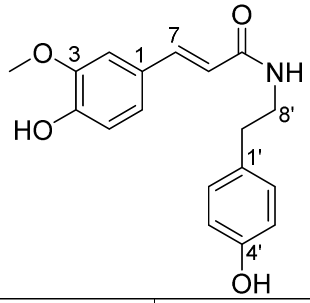

	δC [1]	δH [2]
Feruloyl		
1	131.30, C	
2	111.55, CH	7.12 (1H, d, *J* = 1.9)
3	149.29, C	
4	149.85, C	
5	116.47, CH	6.79 (1H, d, *J* = 8.4)
6	123.21, CH	7.00 (1H, dd, *J* = 8.4, 1.9)
7	142.01, CH	7.43 (1H, d, *J* = 15.7)
8	118.75, CH	6.40 (1H, d, *J* = 15.7)
9	169.17, C	
3-OCH_3	56.39, CH_3	3.88 (3H, s)
Tyramine		
1′	128.28, C	
2′	130.72, CH	7.06, (1H, d, *J* = 8.5)
3′	116.26, CH	6.72 (1H, d, *J* = 8.5)
4′	156.94, C	
5′	116.26, CH	6.72 (1H, d, *J* = 8.5)
6′	130.72, CH	7.06 (1H, d, *J* = 8.5)
7′	42.54, CH_2	2.75 (1H, d, *J* = 7.4)
8′	35.81, CH_2	3.47 (1H, d, *J* = 7.4)

[1] Measured in CD_3OD with 62.5 MHz. [2] Measured in CD_3OD with 250 MHz.

Table S3. DPPH radical scavenging activity of **1**-**3.**

Concentration (μg/mL)	DPPH Radical Scavenging (%)			
	L-Ascorbic Acid	1	2	3
500	- [1]	101.99 ± 0.37	8.66 ± 0.30	97.23 ± 0.33
250	98.14 ± 0.15	100.78 ± 1.46	7.28 ± 0.80	95.51 ± 0.10
100	97.03 ± 0.26	89.88 ± 1.25	3.89 ±2.50	93.65 ± 1.70
50	97.28 ± 0.27	85.92 ± 1.27	3.78 ± 1.40	78.51 ± 0.06
25	97.50 ± 0.53	82.94 ± 0.56	3.61 ± 1.11	70.53 ± 1.74
10	98.01 ± 0.07	51.54 ± 1.30	4.43 ± 0.36	63.25 ± 0.83
5	97.41 ± 0.46	38.70 ± 2.24	3.58 ± 1.79	54.29 ±0.30
2	87.36 ± 2.24	24.91 ± 0.78	2.73 ± 2.15	14.90 ± 1.63
1	45.09 ± 1.40	11.87 ± 0.82	2.93 ± 2.39	0.86 ± 2.12

[1] Not tested.

Table S4. ABTS radical scavenging activity of **1**-**3.**

Concentration (μg/mL)	ABTS Radical Scavenging (%)			
	L-Ascorbic Acid	1	2	3
50	99.77 ± 0.08	66.38 ± 1.47	8.71 ± 0.21	82.19 ± 1.38
25	99.75 ± 0.06	66.31 ± 1.84	3.97 ± 0.43	78.71 ± 0.06
5	70.41 ±0.28	27.97 ± 4.11	2.03 ± 0.00	44.18 ± 2.64

Table S5. Pancreatic lipase inhibition by **1**-**3.**

Concentration (μg/mL)	Inhibition of Pancreatic Lipase (%)			
	Orlistat	1	2	3
100	- [1]	41.11 ± 0.01	52.65 ± 0.00	17.28 ± 0.02
50	- [1]	37.95 ± 0.02	42.68 ± 0.01	16.96 ± 0.02
25	- [1]	21.17 ± 0.01	36.64 ± 0.02	15.87 ± 0.02
10	- [1]	0.73 ± 0.01	15.07 ± 0.01	14.76 ± 0.01
1.00	54.53 ± 0.02	- [1]	- [1]	- [1]
0.50	51.17 ± 0.01	- [1]	- [1]	- [1]
0.25	27.63 ± 0.01	- [1]	- [1]	- [1]
0.10	22.98 ± 0.01	- [1]	- [1]	- [1]

[1] Not tested.

Table S6. Spiked qHNMR data of compound **1** in EA-a.

	% w/w of Compound 1 in EA-a Sample	Amount of Compound 1 (mg)	Spiked Amount of 1 (mg)	% w/w of Compound 1 in Spiked EA-a Sample	Amount of Compound 1 in Spiked Sample (mg)	Amount of Spiked Compound 1 in Spiked Sample (mg)
EA-a 1	4.35	0.435		9.35	0.935	0.500
EA-a 2	4.76	0.465	0.500	9.79	0.989	0.524
EA-a 3	4.83	0.488		9.57	0.967	0.479
Average	4.65	0.463	0.500	9.57	0.963	0.501
S.D.	0.26	0.027	-	0.22	0.027	0.023
C.V.	0.06	0.058	-	0.02	0.028	0.045

Table S7. qHNMR data of compounds **2** and **3** in MC-bg.

	% w/w of Compound 2 in EA-a sample	Amount of Compound 2 (mg)	% w/w of Compound 3 in EA-a Sample	Amount of Compound 3 (mg)
MC-bg 1	0.87	0.087	4.64	0.464
MC-bg 2	0.86	0.086	4.60	0.460
MC-bg 3	0.88	0.088	4.58	0.458
Average	0.87	0.087	4.61	0.461
S.D.	0.01	0.001	0.03	0.003
C.V.	0.01	0.011	0.01	0.007
